# Perspectives and Preferences of Adult Smartphone Users Regarding Nutrition and Diet Apps: Web-Based Survey Study

**DOI:** 10.2196/27885

**Published:** 2021-07-30

**Authors:** Maria F Vasiloglou, Stergios Christodoulidis, Emilie Reber, Thomai Stathopoulou, Ya Lu, Zeno Stanga, Stavroula Mougiakakou

**Affiliations:** 1 ARTORG Center for Biomedical Engineering Research University of Bern Bern Switzerland; 2 MICS Laboratory Biomathematics Research Group CentraleSupélec, Gif-sur-Yvette Paris France; 3 Department of Diabetes, Endocrinology, Nutritional Medicine and Metabolism Inselspital Bern University Hospital, University of Bern Bern Switzerland; 4 Department of Emergency Medicine, Inselspital, Bern University Hospital University of Bern Bern Switzerland

**Keywords:** dietary assessment, end-users, mHealth, mobile apps, smartphone, survey, apps, nutrition, diet, mobile health, users, behavior, behavior change

## Abstract

**Background:**

Digital technologies have evolved dramatically in recent years, finding applications in a variety of aspects of everyday life. Smartphones and mobile apps are being used for a steadily increasing number of tasks, including health monitoring. A large number of nutrition and diet apps are available, and some of them are very popular in terms of user downloads, highlighting a trend toward diet monitoring and assessment.

**Objective:**

We sought to explore the perspectives of end users on the features, current use, and acceptance of nutrition and diet mHealth apps with a survey. We expect that this study can provide user insights to assist researchers and developers in achieving innovative dietary assessments.

**Methods:**

A multidisciplinary team designed and compiled the survey. Before its release, it was pilot-tested by 18 end users. A 19-question survey was finally developed and was translated into six languages: English, German, French, Spanish, Italian, and Greek. The participants were mainly recruited via social media platforms and mailing lists of universities, university hospitals, and patient associations.

**Results:**

A total of 2382 respondents (1891 female, 79.4%; 474 male, 19.9%; and 17 neither, 0.7%) with a mean age of 27.2 years (SD 8.5) completed the survey. Approximately half of the participants (1227/2382, 51.5%) had used a nutrition and diet app. The primary criteria for selecting such an app were ease of use (1570/2382, 65.9%), free cost (1413/2382, 59.3%), and ability to produce automatic readings of caloric content (1231/2382, 51.7%) and macronutrient content (1117/2382, 46.9%) (ie, food type and portion size are estimated by the system without any contribution from the user). An app was less likely to be selected if it incorrectly estimated portion size, calories, or nutrient content (798/2382, 33.5%). Other important limitations included the use of a database that does not include local foods (655/2382, 27.5%) or that may omit major foods (977/2382, 41%).

**Conclusions:**

This comprehensive study in a mostly European population assessed the preferences and perspectives of potential nutrition and diet app users. Understanding user needs will benefit researchers who develop tools for innovative dietary assessment as well as those who assist research on behavioral changes related to nutrition.

## Introduction

To optimally quantify food intake, it is essential to assess nutritional risk, understand dietary patterns, and identify nutrition-related health problems. Accuracy and efficiency in tracking dietary intake requires tools that are validated and easy to use, as conventional methods of assessing diets are prone to errors [1].

Digital technologies, particularly the use of mobile apps, have evolved dramatically in recent years. An estimated 3.7 billion app downloads were performed in 2017. At that time, there were 325,000 mobile health (mHealth) apps available in app stores related to health and fitness [2]. Data collected by smartphones help users improve their self-management and assist behavior changes. Moreover, mobile apps enhance communication between customers and health care professionals, reduce health costs, and improve the dissemination of public health information [3].

The large number of nutrition and diet apps and their numbers of downloads indicate that there is great interest in diet monitoring and assessment [4]. Nutrition and diet apps are among the most widely mHealth used services to promote a healthy lifestyle. Image-based apps that use artificial intelligence (AI) and computer vision are able to recognize the type of food, segment the different parts of the meal, and accurately calculate a meal’s energy content, macronutrients, and partial micronutrients [5,6]. A wide variety of factors influence the uptake and impact of mHealth services, which are related both to the individual person (eg, health-related goals) and the technology (eg, usability and accuracy) [6]. To be able to efficiently use those apps, users need to be able to assess them. However, comparing and evaluating these apps is difficult [6].

Nutrition and diet app engagement relies on several elements, such as the time and effort required to manually track food intake [7]. Although some attempts have been made to reduce the effort involved in taking food records (eg, digital scales) [8] or with the list of food items in nutrient databases [9], these features have not yet been integrated into commercially available nutrition and diet apps.

There are few literature reports on the characteristics and preferences of potential nutrition and diet app users. Social norms appear to play a significant role in adopting nutrition and diet apps, as more technically skilled individuals are generally more likely to engage with digital media [10]. Lee et al [11] reported that five factors played a major role in predicting intention to continue using nutrition and diet apps, namely, recordability, networkability, credibility, comprehensibility, and trendiness. A survey of college-aged individuals in the United States investigated their perspectives on health and fitness apps, and it was reported that the respondents valued the low cost and simplicity of the apps as well as the enjoyment of using them [12]. A review on determining the components that facilitate user engagement with digital health interventions to encourage behavior change and weight management showed how crucial it is to incorporate user perspectives from a very early stage of app development to promote app engagement [13].

There are very few broad studies that investigate users’ opinions on nutrition and diet apps. To increase acceptance and adoption of those apps, it is necessary to gain insight into user perspectives on nutrition and diet apps while encouraging engagement for continued use. In this study, we aimed to explore the perspectives of users on the features, current use, and acceptance of nutrition and diet apps. To the best of our knowledge, this is the most comprehensive nutrition and diet app user survey, both in sample size and questionnaire detail.

## Methods

### Survey

We performed a web-based quantitative survey to collect data on nutrition and diet apps and how users perceive them. We adopted the process of drafting, reviewing, and finalizing the questionnaire used in another survey, and the precise procedure can be found in an earlier paper [14]. The following is a brief description of the basic steps taken.

Based on a thorough literature review of surveys on mHealth apps used for dietary monitoring and assessment, we developed 22 preliminary questions. These questions covered basic demographic information, current use of nutrition and diet apps, criteria for nutrition and diet app selection, and barriers to using these apps. Furthermore, respondents were asked to state their opinion on the importance of specific features as well as their preferences for logging meals and how the results were presented. Next, an interdisciplinary team of experts in AI, computer scientists, dietitians, physicians, pharmacists, and psychologists reviewed the questions and made suggestions for revision.

The survey was submitted for a pilot test to determine whether it was simple, clear, concise, and user friendly. The group of 24 users invited to this pilot survey comprised 11 colleagues from the ARTORG Center for Biomedical Research (nonmembers of the research group) and 13 members of the general public. Of these, 75% (18/24) agreed to take part in the survey. All the participants held a BSc degree, their age range was 22-41 years, and they were familiar with app use. They were asked to provide feedback on the structure, content, readability, flow of questions, and duration of the survey. The interdisciplinary team revised the survey based on the feedback received.

The final survey consisted of 19 questions in English. The survey was also translated into German, French, Italian, Spanish, and Greek by certified translators. It was structured in a multiple-choice format, and data were collected and managed using Research Electronic Data Capture (REDCap); participants took 5-10 minutes to complete the survey.

Details regarding the structure of the survey are described in the following sections. Additionally, a comprehensive Checklist for Reporting Results of Internet E-Surveys (CHERRIES) [15] is provided in Multimedia Appendix 1.

### Inclusion and Exclusion Criteria

Eligible respondents were adults who were able to understand one of the following languages: English, German, French, Italian, Spanish, or Greek. Signed informed consent was required prior to participation. Exclusion criteria included any inability to understand and comply with written and verbal instructions, or inability to give consent.

### Recruitment

The respondents were recruited via (1) social media platforms (ie, Twitter, Facebook), (2) mailing lists of the collaborative universities across Europe, (3) patient associations and foundations, and (4) outpatients of collaborating university hospitals.

### Statistical Analysis

Descriptive statistics were used to present quantitative data (only from completed surveys). A chi-square test of independence was performed to examine the differences between groups. Multiple binary logistic regressions were performed to explain relationships between independent variables (eg, age, sex, education, BMI) and categorical dependent variable (ie, users who used a nutrition and diet app). The results were expressed as odds ratios (ORs) with 95% confidence intervals representing the odds that a participant will use a nutrition and diet app based on their aforementioned variables compared to the odds that they will not use it. Statistical significance was indicated with *P*=.04. RStudio, version 1.0.153 (RStudio PBC) was used for the statistical analysis.

### Ethical Approval

The study was reviewed and declared exempt from ethics review by the Cantonal Ethics Committee, Bern, Switzerland (KEK 2019-00102).

## Results

A total of 3587 people accessed the survey link, and 2399 completed the survey (66.9%). After 17 respondents from were eliminated from the sample for not providing informed consent, data from 2382 respondents (2382/3587, 66.4%) were included in our analyses. The vast majority of the respondents were from Europe (2333/2382, 97.9%). The demographic characteristics of the respondents are provided in Table 1.

**Table 1:** Demographic characteristics of the survey respondents (N=2382).

Characteristic			Value
**Sex, n (%)**			
	Female		1891 (79.4)
	Male		474 (19.9)
	Neither/prefer not to disclose		17 (0.7)
**Age (years)**			
	Average (SD)		27.2 (8.5)
	**Range, n (%)**		
		18-29	1770 (74.3)
		30-39	413 (17.3)
		40-49	111 (4.7)
		50-59	64 (2.7)
		≥60	24 (1.0)
**BMI (kg/m^2^), n (%)**			
	18.5-24.9 (normal weight)		1759 (73.8)
	25-29.9 (overweight)		392 (16.5)
	<18.5 (underweight)		111 (4.7)
	30-34.9 (class I obesity)		82 (3.4)
	35-39.9 (class II obesity)		30 (1.3)
	>40 (class III obesity)		8 (0.3)
**Smoker, n (%)**			
	No		2014 (84.5)
	Yes		349 (14.7)
	No answer		19 (0.8)
**Highest educational level, n (%)**			
	Bachelor’s degree		958 (40.2)
	High school/apprenticeship		744 (31.2)
	Master’s degree		512 (21.5)
	PhD		114 (4.8)
	Other		41 (1.7)
	Primary/intermediate School		8 (0.3)
	No schooling completed		5 (0.2)
**Diseases or conditions,^a^ n (%)**			
	No illnesses or health problems		1495 (62.8)
	Overweight/obesity		194 (8.1)
	Lactose intolerance		138 (5.8)
	Inflammatory bowel syndrome		137 (5.8)
	Anemia		105 (4.4)
	Food allergy		105 (4.4)
	Eating disorders		93 (3.9)
	Acid reflux		92 (3.9)
	High total cholesterol		62 (2.6)
	Other diseases/conditions^b^		322 (13.5)
	No answer		53 (2.2)
**Smartphone user, n (%)**			
	No		154 (6.5)
	Yes		2228 (93.5)
**Operating system, n (%)**			
	Android		1381 (61.9)
	iOS (Apple iPhone)		828 (37.2)
	Windows		4 (0.2)
	I do not know/I do not want to answer		11 (0.5)
	Other		4 (0.2)

^a^Multiple answers could be selected.

^b^Any disease/condition with a prevalence of <2.5% was summed in “Other diseases/conditions.”

### Tracking of Food Intake

It was reported that 29.1% (693/2382) of the respondents tracked their food intake and their tracking methods. Asked about which methods they used, 40.4% (280/693) declared that they used apps, 36.4% (252/693) chose paper and pencil methods, and 22.1% (153/693) preferred other methods. Moreover, 8.2% (57/695) tracked their food by photos/videos, and 4% (28/700) did not express their opinion.

### Use of Nutrition and Diet Apps

In total, 51.5% (1227/2382) of the respondents had used a nutrition and diet app. Among these, 20.1% (247/1227) used the app daily, and 8.3% (102/1227) mentioned using the app only for specific foods or beverages. We also asked how the users identified those apps. Most of the respondents reported finding them via the App Store/Google Play store (467/1227, 38.1%), via social media (192/1227, 15.6%), or on a friend’s recommendation (156/1227, 12.7%). Only 2% (25/1227) found the apps through their dietitian, 0.7% (8/1227) found them through their medical doctor, and 0.5% (6/1227) expressed no opinion. Finally, 5% (59/1227) mentioned other sources, such as recommendations on the web or from their fitness trainers. The 2 most popular apps were MyFitnessPal (239/1227, 19.5%) and Yazio (184/1227, 15%). Both apps are primarily used as calorie counters.

### Reasons for Not Having Used a Nutrition and Diet App

When respondents were asked about the reasons for never having used a nutrition and diet app, the majority (667/1155, 57.8%) indicated that they were not interested, and approximately one-third (340/1155, 29.4%) considered nutrition and diet apps to be too time consuming. Other frequently mentioned reasons were lack of awareness of the apps’ existence (194/1155, 16.8%) and privacy as well as security concerns (184/1155, 15.9%). A few respondents preferred paper and pencil methods (44/1155, 3.8%). Free text responses provided additional reasons for nonuse, which included the strong focus of the currently available apps on weight loss and the fear of developing eating disorders. Of those who had not used a nutrition and diet app (1155/2382), 40.3% (465/1155) were optimistic about trying one in the future, 49.6% (573/1155) were negative, and 10.1% (117/1155) did not express their opinion.

### Associations Between Different Variables and Nutrition and Diet App Use

Nutrition and diet app users and nonusers differed significantly by gender (*P*<.001), BMI (*P*<.001), and educational level (*P*=.003). Logistic regression, when adjusted for gender, BMI, and educational level, indicated that the odds that an individual used a nutrition and diet app were 2.5 times greater for male participants compared to female participants (OR 2.45, 95% CI 1.96-3.06; *P*<.001).

Based on the participants’ self-reported weight and height, we described the distribution of nutrition and diet app use according to the World Health Organization classification of BMI. Thus, 54.1% (60/111) of participants in the underweight category, 54.2% (954/1759) of those in the normal weight category, 44.6% (175/392) of those in the overweight category, and 31.7% (38/120) of those in the obese category reported that they had used nutrition and diet apps. According to the logistic regression model mentioned above, overweight reduces the likelihood of using nutrition and diet apps compared to normal weight (OR 0.59, 95% CI 0.47-0.74; *P*<.001). Individuals with class I or II obesity are less likely to use an app than individuals with normal weight (obese class I: OR 0.42, 95% CI 0.26-0.67, *P*<.001; obese class II: OR 0.27, 95% CI 0.1-0.62, *P*=.03).

**Figure 1:**
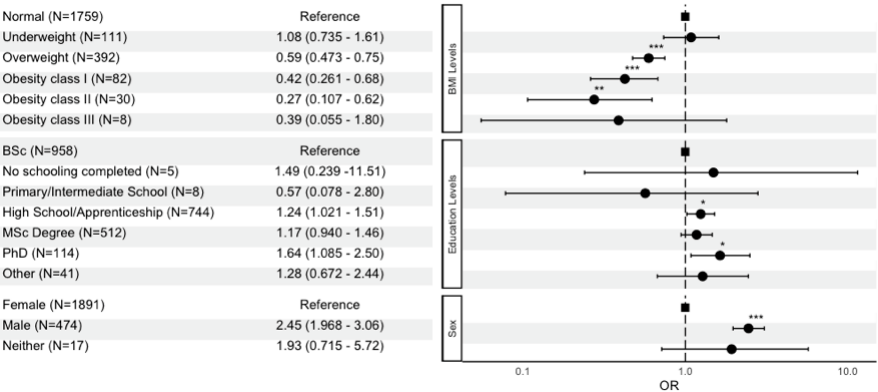
Forest plot showing the influence of BMI, educational levels, and sex on the use of nutrition and diet apps. *P<.05; **P<.01; ***P<.001.

With regard to the level of education, the logistic regression model showed that having completed only high school or having obtained a PhD increased the probability of using a nutrition and diet app (high school: OR 1.24, 95% CI 1.02-1.51, *P*=.03; PhD: OR 1.64, 95% CI 1.08-2.49, *P*=.02). Figure 1 shows the results of the logistic regression for the variables in relation to the use of nutrition and diet apps.

We also investigated the respondents who were living with a disease or health condition and had used a nutrition and diet app. We focused on the most prominent diseases and conditions in our sample (obesity/overweight, lactose intolerance, irritable bowel syndrome [IBS], anemia, food allergy, eating disorders, acid reflux, high cholesterol levels). Only 32.5% (63/194) of people who declared they were living with obesity or overweight were using nutrition and diet apps. The percentage of people who used nutrition and diet apps and were affected by lactose intolerance was 42.8 (59/138), with 31.4% (43/137) for people with IBS, 32.4% (34/105) for people with anemia, 42.9% (45/105) for people with food allergies, 13% (12/93) for people with eating disorders, 38% (35/92) for people with acid reflux, and 47% (29/62) for people with high cholesterol levels. According to the logistic regression model between app users and each disease, individuals with overweight/obesity, IBS, anemia, or eating disorders were less likely to use a nutrition and diet app than individuals who did not have the respective conditions. The ORs for the most prominent diseases are provided in detail in Table 2.

**Table 2:** Logistic regression of nutrition-related diseases and the use of nutrition and diet apps.

Disease	Odds ratio (95% CI)
Overweight/obesity* (n=194)	0.49 (0.34-0.70)
Lactose intolerance (n=138)	0.90 (0.61-1.32)
Irritable bowel syndrome** (n=137)	0.58 (0.38-0.86)
Anemia** (n=105)	0.49 (0.31-0.77)
Food allergy (n=105)	0.88 (0.56-1.35)
Eating disorders* (n=93)	0.16 (0.08-0.29)
Acid reflux (n=92)	0.82 (0.51-1.30)

*P<.001.

**P<.01.

### Criteria for Selecting and Reasons for Not Selecting a Nutrition and Diet App Among the Whole Sample

A detailed overview of the criteria for selecting a nutrition and diet app is provided in Table 3. The most prominent criteria were that the app was easy to use, was free of charge, supported automatic calorie/nutrient estimation, and integrated automatic food recording (eg, bar code readers, meal images).

Asked to indicate what they considered to be barriers to selecting a nutrition and diet app, participants reported that they would not choose such an app if major foods were missing, if it gave incorrect estimations of calories or nutrients, if local foods were not supported, or if the estimation of portion size was not accurate. They also emphasized the vital role of personalization (language, measurement units, etc) in the apps. A detailed list of the barriers and their selection frequencies are provided in Table 3.

**Table 3:** Criteria for selecting and reasons for not selecting nutrition and diet apps (N=2382).

Criteria and barriers		Value, n (%)
**Criteria for selecting a nutrition and diet app**		
	Easy to use/convenient	1570 (65.9)
	Costless/free of charge	1413 (59.3)
	Automatic calorie estimation is supported	1231 (51.7)
	Automatic nutrient estimation is supported	1117 (46.9)
	Automatic food recording is supported	1115 (46.8)
	Validated and certified	1036 (43.5)
	Self-explanatory (no need for training)	953 (40)
	History records are supported	755 (31.7)
	Recipe and menu import functions are supported	729 (30.6)
	Provides an option for adding nutritionally related events (eg, reflux)	443 (18.6)
	A link for data transmission (eg, other apps) is supported	217 (9.1)
	No opinion	195 (8.2)
	Other	95 (4)
**Barriers to selecting a nutrition and diet app**		
	Major foods are missing	977 (41.0)
	Incorrect calorie and nutrient estimation	798 (33.5)
	Local foods are not supported	655 (27.5)
	Unconvincing portion size estimation	648 (27.2)
	Not personalized	503 (25.1)
	Not validated and not certified	567 (23.8)
	No opinion	522 (21.9)
	Only manual entry of food type is allowed	464 (19.5)
	Incorrect automatic food item recognition	448 (18.8)
	Recipes are not considered for nutrient estimations	376 (15.8)
	Only manual entry of portion size is allowed	345 (14.5)
	User is required to be tech savvy	310 (13.0)
	History records are not supported	295 (12.4)
	Sharing of history records is not supported	88 (3.7)
	Other	83 (3.5)

### Importance of Specific Features in Nutrition and Diet App Selection

**Figure 2:**
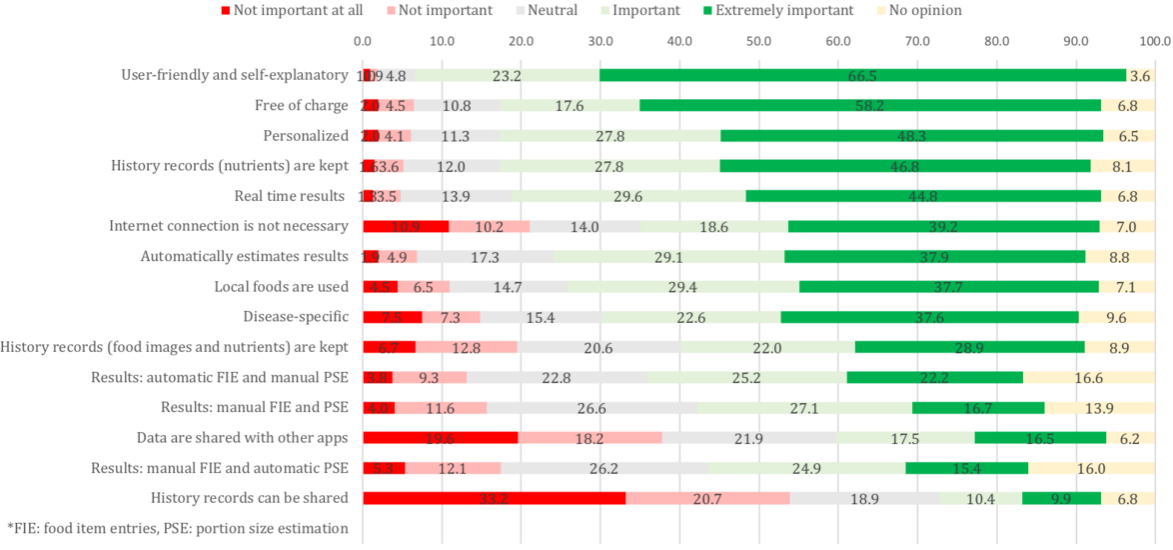
Importance of features of nutrition and diet apps (%).

Asked to rate the importance of specific features for a nutrition and diet app on a scale from 1 (not important at all) to 5 (extremely important), users considered user-friendliness, self-explanatory nature of the app, provision of real-time results, and the app being free of charge as important characteristics (Figure 2). Moreover, the automatic estimation and recording of results was judged as important. On the other hand, respondents ranked the ability to share history records and the fact that the data could be shared with other apps as least important.

### Comparing the Criteria for and Barriers to Nutrition and Diet App Use of Users and Nonusers

We also investigated possible differences between the users and nonusers of nutrition and diet apps. In fact, both groups mentioned that their top 3 reasons for selecting a nutrition and diet app were free cost, ease of use, and integration features that automatically estimated energy and nutrient content. The main difference identified was that nutrition and diet app users stated that validation of the apps was among the top 5 important criteria for choosing an app, while nonusers mentioned that automatic food recording would be of great importance if it were supported by nutrition and diet apps.

In terms of barriers, both groups reported that they would not choose an app with incorrect energy and nutrient estimates that did not provide correct portion sizes. Regarding differences, app users viewed not including the most important foods in the app’s nutritional database as a major barrier. In contrast, nonusers stated that lack of personalization and lack of inclusion of local foods in the nutrient database would be their greatest barriers.

### Preferences Regarding On-screen Display of Nutrient Content and Time Required to Capture Data

When respondents were asked about their preferences regarding on-screen display of the calorie estimations of a meal, 43.3% (1031/2382) indicated that they would prefer to receive the exact value. Regarding the on-screen display of macronutrients, 26.6% (632/2382) would prefer they be presented as a traffic light system (green for low, yellow for moderate, red for high), and 26.5% (631/2382) favored the display of either an accurate value or a combination of a traffic light system and accurate values.

The respondents were also asked about their preferences for the display of the amount of food or drink; they were mostly in favor of values (eg, grams, milliliters) (1284/2382, 53.9%), while a considerable number of respondents (784/2382, 32.9%) preferred common household measures (eg, cups, spoons). Fewer respondents (191/2382, 8%) preferred an abstract portion size (small, medium, large), and 5.2% (124/2382) expressed no opinion.

When asked about the relationship between the response time and the accuracy of the results, respondents were given the following choices: (1) taking 2 photos and obtaining results within 5 seconds that were as accurate as results from a dietitian, (2) taking just 1 photo and obtaining results in real time that were less accurate than those from a dietitian, and (3) recording a video and obtaining results after 5 seconds that were more accurate than those from a dietitian. Most of the respondents preferred the second option (1305/2382, 54.8%), followed by the third (721/2382, 30.3%) and the first (354/2382, 14.9%).

## Discussion

### Principal Findings

We investigated users’ perspectives and preferences regarding nutrition and diet apps. Approximately half of our respondents (1227/2382, 51.5%) reported having used a nutrition and diet app. These results are consistent with data obtained among 1191 respondents in a study investigating weight-management apps in Saudi Arabia, in which 43.1% (513/1190) of the respondents had used weight management apps [16]. A similar cross-sectional survey by Krebs et al [17] among US mobile phone users (n=1604) found that 58.2% (934/1604) of the respondents had downloaded a health-related mobile app.

In our study, the most frequently chosen criteria for selecting a nutrition and diet app were ease of use (1570/2382, 65.9%); lack of cost (1413/2382, 59.3%); automatic energy estimation (1231/2382, 51.7%); automatic nutrient estimation (1117/2382, 46.9%); and automatic food recording (1115/2382, 46.8%). These results reflect those of several studies that investigated the usability of apps as a valuable factor and that concluded that complex and difficult-to-use apps would not be preferred [16-21]. In a study conducted by König et al [7] in 2018 regarding the adoption of nutrition and fitness apps, it was stated that the decision to use an app may be influenced by whether the data collection is active or passive. For instance, fitness apps that gather data automatically have better use rates than apps with manual data entry [7]. In terms of automatic (ie, AI) features in nutrition apps, however, despite the implied support from the AI technology to yield easy-to-use apps, [4] an analysis of popular nutrition apps (n=13) found no application of AI technologies (eg, image recognition or natural language processing) in those apps.

### Issues of App Accuracy and Food Databases as Barriers

One-third of the participants (794/2382) in our study mentioned that incorrect nutrient and energy output would be a barrier to selecting a specific nutrition and diet app. This is also in accordance with observations from another study, which showed that the accuracy and trustworthiness of these apps are important. Respondents expressed concerns stemming either from the inherent characteristics of the apps, such as whether they were developed by experts, or from uncertainty coming from the user (eg, human errors of forgetting) [18]. In fact, in a study that aimed to analyze common user errors when using an image-based app, 12.8% of the acquired images had to be discarded due to mistakes in the capturing procedure [22].

Our study also revealed that respondents would not select apps that had issues related to their food and nutrient databases, such as absence of major or local foods and incorrect estimations of calories or nutrients. Respondents in a qualitative study for weight management (n=24) considered that larger databases were more convenient and easier to use than smaller databases, even though they had difficulty identifying the right foods among the numerous food options [13]. Another database-related barrier reported by some respondents was that of missing foods (ie, ethnic/traditional foods) and misreported content (ie, provision of content of calories but not of macronutrients) in the databases [21]. The same concern was reported by users in another study, in which some respondents expressed doubts about the accuracy of nutrient databases and others expressed concerns about incorrect micronutrient calculations [21]. Moreover, in a qualitative study exploring the experiences of mHealth app use by young adults (n=19), it was mentioned that respondents were doubtful about the reliability of the portion size estimation, which may lead to lower confidence in monitoring intake [23].

### User Preferences Concerning Nutrient and Energy Output of Nutrition and Diet Apps

Participants in our study preferred the display of energy and macronutrient content over other options, the former as an accurate value and the latter as an accurate value or traffic light system. In a study of nutrition and diet apps in China, it was found that the most frequently provided output was energy (38/44, 86%); however, none of the detected apps provided any information on macronutrients or salt [24], [23]. Mixed results were found in a study of 24 healthy volunteers, in which color coding was found to be effective by some respondents, while others considered that this approach might promote negative feelings. Some female respondents (n=8) mentioned that they were concerned because the apps might make them compulsive about using them. It was implied that these apps could lead to the development of eating disorders, as they mostly target calories and body weight [21].

In our study, some respondents indicated that current apps focus largely on weight loss rather than on behavioral change, implying a heightened risk that app users could develop an eating disorder. The dynamic nature of weight loss was discussed in [24]. The authors considered that apps can contribute to the development or exacerbation of eating disorders but can also be used to treat these disorders.

### Reasons for Not Having Used Nutrition and Diet Apps

The majority of the 48.5% (1155/2382) self-declared nonusers of nutrition and diet apps in our survey said they did not use them due to lack of interest. Approximately one-third of nonusers believed that nutrition and diet apps are time-consuming, and smaller proportions reported lack of knowledge of their existence as well as lack of trust as reasons for nonuse. These findings are in line with a survey conducted among 1604 US citizens, in which 42% of respondents (674/1604) reported never having used a health app. Among these nonusers, the main reasons for nonuse were lack of interest, high app cost, and lack of need [17].

Furthermore, in a qualitative study on health apps conducted among 20 adolescents, the main barriers to using the apps included unfamiliarity with app functionalities and ignorance of their existence. In general, many adolescents did not consider health management as a high priority among their interests [19]. Similarly, another qualitative study [20] examined user perspectives (44/77 participants, 57%, owned health apps) in terms of the content elements of health apps that encourage or hinder their use. Those who did not use apps mentioned impeding factors that included unfamiliarity with health apps (8/33, 25%). Additionally, in corroboration of our findings, some respondents indicated that they had no need for health apps, either because they were using other tools such as websites or because they did not consider themselves in need of such an app because they had already adopted healthy habits. In the same study, a lack of app literacy was noted in that participants did not know which apps were considered good or did not know how to use them [20].

In our study, we found that people with specific nutrition-related diseases, such as obesity, IBS, anemia, or eating disorders, were less likely to use a nutrition and diet app than individuals without the respective conditions. Although we had expected the opposite outcome, we can only speculate that this finding can be attributed to unfamiliarity with the available apps or possible difficulties in finding an app that is dedicated to their condition or is trustworthy. Further research is needed to confirm those findings and explore the aforementioned contradiction.

### Where Do Users Hear About or Find Nutrition and Diet Apps?

Slightly over half of the participants in our study reported having used nutrition and diet apps. The majority found them by searching app stores, reading about them on social media, or receiving a recommendation from a friend. Similar findings from another study [17] revealed that respondents learned about the apps from app stores (327/934, 35%) or from friends or family (287/934, 30.7%), but only 20.37% (210/1031) learned about the apps from a physician’s recommendation [17]. Our results match those observed by Peng et al [20], who found that respondents were recommended a health app via a family member or friend; this suggests a substantial social influence. Analogously, the same outcome was found in a web-based survey, in which the participants selected apps based on recommendation from friends or family (154/513, 30%) or from social media influencers (93/513, 18.1%) [16].

### Comparison of Criteria and Barriers for App Users in the General Public and Health Care Professionals Who Recommend Nutrition and Diet Apps

A survey of health care professionals (n=1001) [14] showed that they would choose an app if it were easy to use (872/1001, 87.1%) and free of charge (727/1001, 72.6%). However, they also prioritized validation of the app (682/1001, 68.1%) and only then considered the importance of an automatic system for recording food (566/1001, 56.5%), followed by automatic nutritional estimation (525/1001, 52.4%). In terms of barriers, health care professionals agree with users from the general public that food database inaccuracy, missing food items, and lack of personalization are critical issues. However, the fourth criterion for health care professionals was technical knowledge (433/1001, 43.3%), whereas few (310/2382, 13%) end users mentioned that technical knowledge would hinder their use of such apps.

### Perception of Obesity

Approximately one-fifth of individuals in our study (476/2382) were classified as either overweight or obese based on BMI calculations of self-reported weight and height. However, when the individuals were asked if they had any health or medical conditions, only 8.1% (194/2382) declared that they were obese or overweight, thus showing a possible weight misperception. Data from the National Health and Nutrition Examination Survey of 4784 individuals living with overweight or obesity showed that 71% (3397/4784) of the participants misperceived their weight. This misperception was associated with lower likelihood of interest in weight loss and less physical activity [25]. Moreover, another study found that people can report their weight and height with reasonable accuracy, but most people with obesity do not consider themselves as obese. It was also noted that adults with obesity who were unable to correctly classify themselves as such may neglect health messages related to obesity and lack motivation to lose weight [26].

### Strengths and Limitations

This is the largest study to date that documents the perspectives of European citizens in relation to nutrition and diet apps. These results add to the rapidly expanding field of apps in dietary monitoring and assessment by enhancing the understanding of the needs of users; thus, it creates a clearer idea of their preferences. Another strength is that the survey was made available in 6 different languages, namely, English, Spanish, German, French, Italian, and Greek, which has not been implemented in any other study on this topic. Furthermore, we aimed to minimize bias in terms of the investigators' professions. For this reason, the study was designed by a multidisciplinary team that represented diverse opinions from different scientific fields. Moreover, the distribution of the survey was not restricted to specific user groups but aimed to reach a general population sample. In addition, the distribution of the survey was not limited to social media but was also extended to mailing lists of collaborating universities, patient associations and foundations, and outpatients of collaborating university hospitals.

As a potential limitation, users with greater interest in nutrition apps or those with prior experience using them may have introduced self-selection bias, as they were more likely to participate in the survey. Our sample consisted to a large extent of Swiss and Greek persons; therefore, we cannot generalize our findings to the whole European population. Another drawback is that the survey was limited to people who had internet access. Additionally, in our study, 4.7% (112/2382) of the respondents were underweight, 73.8% (1758/2382) were in the normal weight range, 16.5% (393/2382) were overweight, and 5% (119/2382) had obesity. However, the findings of the current study do not support the European data. More specifically, the average BMI in the European Union population in 2014 was as follows: 2.3% underweight, 46.1% normal weight, 35.7% overweight, and 15.9% obese [27]. Given the convenience sampling that was adopted, the generalizability of these findings may not represent the distribution of nutrition and diet app users.

### Future Work

The insights gained from this study may provide a foundation for further studies that will include a broader sample size consisting of larger percentages of participants from different European countries. Larger studies are needed to be able to draw reliable conclusions on the overall opinions of smartphone users on nutrition and diet apps. Interpreting user perspectives on nutrition and diet apps provides important clues for app development and improvement. However, intervention studies are needed to test the usability of the apps as well as whether this theory-based information could lead to persistent nutrition and diet app use.

Future apps that focus on eating disorder recovery should explore the different types of feedback in terms of visualization. This is an important aspect in that colors may considerably impact users’ emotional responses, and red and green patterns seem to promote negative behaviors [28]. Weight loss and calorie counting should be treated with caution, because patients may engage in compulsive logging; accurate macronutrient and energy tracking may then encourage unhealthy diets or disordered eating behavior.

Finally, in our study, only 1% (25/2382) of respondents reported that an app was recommended to them by their dietitian. Because nutrition and diet app technology is pervasive, continuous professional development is crucial for health care professionals, especially dietitians, who are responsible for assessing people's nutritional status. Therefore, these professionals should be well informed and keep up to date to be able to suggest reliable apps to their clients and patients. Thereby, the apps would act as invaluable tools for better self-management and dietary monitoring, while the users would be aware of which apps they should avoid.

### Conclusions

This comprehensive study in a mostly European population assessed the preferences and perspectives of potential nutrition and diet app users. The findings suggest that users would select nutrition and diet apps that are easy to use, are free of charge, and automatically estimate the energy and nutrient content of foods with automatic food recording capabilities. Significant barriers to selecting a nutrition and diet app include inaccurate food databases that omit key foods, inaccurate energy and nutrient estimations, and lack of validation and personalization. Understanding user needs will benefit both researchers who develop tools for innovative dietary assessment and those who assist research on behavioral changes related to nutrition. Researchers from different fields, such as nutrition, medicine, computer science, and AI, who are involved in nutrition and diet app development need the insight of the user perspective to design and develop apps that meet users’ requirements and needs.

## Appendix

Multimedia Appendix 1 Checklist for Reporting Results of Internet E-Surveys (CHERRIES).

## Abbreviations

AI: artificial intelligence
CHERRIES: Checklist for Reporting Results of Internet E-Surveys
IBS: irritable bowel syndrome
mHealth: mobile health
OR: odds ratio
REDCap: Research Electronic Data Capture
