# Supplementary material for: Perspectives and Preferences of Adult Smartphone Users Regarding Nutrition and Diet Apps: Web-Based Survey Study
Source: JMIR Mhealth Uhealth. 2021 Jul 30;9(7):e27885. doi: 10.2196/27885 (PMC8367144; doi:10.2196/27885)
Supplement: Multimedia Appendix 1 [file mhealth_v9i7e27885_app1.doc]

# Appendix

**Table 1.** Checklist for Reporting Results of Internet E-Surveys (CHERRIES)

| Item Category | Checklist Item | Item |
| --- | --- | --- |
| **Design** | Describe survey design | Convenience sample survey. |
| **IRB (Institutional Review Board) approval and informed consent process** | IRB approval | The study was reviewed and declared exempt from ethics review by the Cantonal Ethics committee, Bern, Switzerland (KEK 2019-00102). |
|  | Informed consent | Signed informed consent was required prior to participation. |
|  | Data protection | The data were used exclusively for the present study and handled with uttermost discretion.  They were only accessible to authorised personnel who require the data to fulfil their duties within the scope of the research project. All paper documents are stored in locked filings cabinets on site, separately from the study data, in the custody of a person who is not involved in  the study. The electronic documents are kept in the REDCap database, which is a working data management system allowing to track any modification to stored data (single logins) and providing back-up functions. |
| **Development and pre-testing** | Development and testing | The survey was submitted for a pilot test to find out whether it was simple, clear, concise, and user-friendly. In total, 18 users evaluated the survey before communicating to the general public. The authors revised the survey based on the feedback received. |
| **Recruitment process and description of the sample having access to the questionnaire** | Open survey versus closed survey | Open survey accessible with a single link and no password. |
|  | Contact mode | Recruitment was performed through dissemination in social Media, mailing lists, patients associations. |
|  | Advertising the survey | The advertisement was solely performed through social Media. |
| **Survey administration** | Web/E-mail | A single link to a REDcap survey was administered through posting it to web pages and by email |
|  | Context | Social media (Linkedin, Facebook, Twitter) mailing lists of universities and university hospitals as well as patients’ associations. |
|  | Mandatory/voluntary | Voluntary |
|  | Incentives | No monetary incentives were given |
|  | Time/Date | Timeframe spanned between 01/06/2020 and 31/12/2020 |
|  | Randomization of items or questionnaires | No |
|  | Adaptive questioning | Adaptive questioning was used in order to reduce the complexity of the questionnaire. |
|  | Number of Items | 19 questions were included in the survey. For each question a number of aspects were collected. In total, 84 REDcap field items/variables constituted the complete questionnaire while 18 of them were conditional. |
|  | Number of screens (pages) | 7 |
|  | Completeness check | All answers were obligatory for the responders. Completeness was required for the final submission of the questionnaire. |
|  | Review step | Not included |
| **Response rates** | Unique site visitor | Not available |
|  | View rate (Ratio of unique survey visitors/unique site visitors) | Not available |
|  | Participation rate (Ratio of unique visitors who agreed to participate/unique first survey page visitors) | Not available |
|  | Completion rate (Ratio of users who finished the survey/users who agreed to participate) | 99.29% (2382 out of 2399) |
| **Preventing multiple entries from the same individual** | Cookies used | No cookies were used |
|  | IP check | No IP check was performed |
|  | Log file analysis | No log file analysis was performed |
|  | Registration | Not required |
| **Analysis** | Handling of incomplete questionnaires | Only complete questionnaires were included in the survey. Incomplete questionnaires were excluded. |
|  | Questionnaires submitted with an atypical timestamp | None |
|  | Statistical correction | Not performed |
